# The burden of oral disorders in Latin America and Caribbean countries from 1990 to 2023 and projections until 2050: a systematic analysis for the Global Burden of Disease Study 2023

**DOI:** 10.1016/j.lana.2026.101517

**Published:** 2026-06-08

**Authors:** GBD 2023 Latin America and Caribbean Oral Disorders Collaborators, Maria Jesus Rios-Blancas, Christian Razo, Linda Morales-Juárez, Ricardo X. Martinez, Lucero Lopez-Lopez, Fernando Neves Hugo, S. Aida Borges-Yáñez, Carol Guarnizo-Herreño, Carolina Hommes, Caroline Stein, Betine P. Moehlecke Iser, Richard Niederman, Escoffié-Ramírez Mauricio, Vanessa Aldaz-Rodriguez, Roberto A. Leon-Manco, Deborah Carvalho Malta, Marco Cornejo-Ovalle, Carlos Garcia-Zavaleta, Paul Nam, Rafael Lozano

**Keywords:** Burden of disease, YLDs, Prevalence, Incidence, Oral disorders

## Abstract

**Background:**

Oral disorders (OD) are the most prevalent non-communicable conditions worldwide. We analyse the burden of OD in Latin America and the Caribbean (LAC) from 1990 to 2023, with projections to 2050.

**Methods:**

This analysis used Global Burden of Disease (GBD) 2023 estimates of incidence, prevalence, and years lived with disability (YLDs) for untreated dental caries of deciduous (UDCD) and permanent teeth (UDCP), severe periodontitis, edentulism, and other OD across 33 LAC countries. DisMod-MR 2.1, spatiotemporal regression, and MR-BRT were applied. YLDs were calculated as prevalence multiplied by disability weights. Associations with the Socio-demographic Index (SDI) were assessed using LOWESS smoothers. Forecasts to 2050 incorporated demographic and risk-factor trends.

**Findings:**

In 2023, OD in LAC caused 290.8 million (95% UI 259.3–325.4) incident cases, 308.2 million (285.9–332.7) prevalent cases, and 2.41 million (1.51–3.48) YLDs. They ranked first in prevalence, second in incidence, and tenth in YLDs among all causes over the past three decades. UDCD peaked in childhood, UDCP in young adulthood and midlife; while severe periodontitis and edentulism predominated in older adults, with YLDs increasing sharply with age. Higher SDI correlated inversely with edentulism but positively with severe periodontal disability. By 2050, YLDs are projected to reach 3.81 million (2.28–5.80).

**Interpretation:**

Despite progress in child oral health, OD remain a leading cause of YLDs in LAC. Integrating oral health into primary care and UHC across the life course, alongside action on upstream social and commercial determinants is essential to reduce the burden.

**Funding:**

The GBD2023 Study is supported by the Gates Foundation.

## Abstract (es)

**Antecedentes:**

Los padecimientos orales (PO) destacan como las enfermedades no transmisibles más prevalentes a nivel mundial. Analizamos la carga de los PO en América Latina y el Caribe (ALC) entre 1990 y 2023, con proyecciones hasta 2050.

**Métodos:**

Este análisis utilizó estimaciones del Estudio de la Carga Global de la Enfermedad 2023 (GBD 2023) sobre incidencia, prevalencia y años vividos con discapacidad (AVD) por caries dental no tratada en dentición decidua (CDD) y permanente (CDP), periodontitis severa, edentulismo y otras EB en 33 países de ALC. Se usaron modelos DisMod-MR 2.1, regresión espacio-temporal (ST-GPR) y MR-BRT. Los AVD se calcularon multiplicando la prevalencia por los ponderadores de discapacidad. Se evaluó la asociación de la carga de EB con el Índice Sociodemographic (ISD) mediante curvas suavizadas LOWESS. Las proyecciones hasta 2050 incorporaron tendencias demográficas y de factores de riesgo.

**Resultados:**

En 2023, se estimaron 290.8 millones (II 95%: 259.3–325.4) de casos incidentes, 308.2 millones (285.9–332.7) de casos prevalentes y 2.41 millones (1.51–3.48) de AVDs por PO en ALC. De 1990 a 2023, las PO ocuparon el primer lugar en prevalencia, el segundo en incidencia y el décimo en AVD entre todas las causas en la región. La CDD fue más frecuente en la infancia, y la CDP en la adultez; mientras que la periodontitis severa y el edentulismo predominaron en adultos de 60 años y más, con un aumento marcado de los AVD con la edad. Un mayor ISD se asoció con menor edentulismo, pero con mayor carga de AVD por periodontitis severa. Para 2050, se proyecta que los AVD alcancen 3.56 millones (2.40–5.07).

**Interpretación:**

A pesar de los avances en la salud bucal infantil, las EB continúan siendo una de las principales causas de AVD en ALC. La integración de la salud bucal en la atención primaria y en la cobertura universal de salud en todas las etapas de la vida, junto con intervenciones dirigidas a los determinantes sociales y comerciales estructurales, es esencial para reducir esta carga.

**Financiamiento:**

El Estudio GBD 2023 fue financiado por la Fundación Gates.

## Resumo

**Antecedentes:**

As doenças orais (DO) constituem as condições não transmissíveis mais prevalentes no mundo. Analisamos a carga das DO na América Latina e no Caribe (ALC) entre 1990 e 2023, com projeções até 2050.

**Métodos:**

Esta análise utilizou estimativas do Global Burden of Disease (GBD) 2023 de incidência, prevalência e anos vividos com incapacidade (YLDs) para cárie dentária não tratada na dentição decídua (UDCD) e permanente (UDCP), periodontite severa, edentulismo e outras DO em 33 países da ALC. Foram aplicadas as ferramentas DisMod-MR 2.1, regressão espaço-temporal e MR-BRT. Os YLDs foram calculados multiplicando a prevalência pelos pesos de incapacidade. As associações com o Índice Sociodemographic (SDI) foram avaliadas por meio de suavização LOWESS. As projeções até 2050 incorporaram tendências demográficas e de fatores de risco.

**Resultados:**

Em 2023, as DO na ALC causaram 290.8 milhões (II 95%: 259.3–325.4) de casos incidentes, 308.2 milhões (285.9–332.7) de casos prevalentes e 2.41 milhões (1.51–3.48) de YLDs. Nas últimas três décadas, ocuparam o primeiro lugar em prevalência, o segundo em incidência e o décimo em YLDs entre todas as causas. A UDCD apresentou pico na infância, na juventude e meia-idade; enquanto a periodontite severa e o edentulismo predominaram em adultos mais velhos, com YLDs aumentando acentuadamente com a idade. Um SDI mais elevado correlacionou-se inversamente com o edentulismo, mas positivamente com a incapacidade associada à periodontite severa. Até 2050, projeta-se que os YLDs alcancem 3.56 milhões (2.40–5.07).

**Interpretação:**

Apesar dos avanços na saúde bucal infantil, as DO permanecem uma importante causa de YLDs na ALC. A integração da saúde bucal na atenção primária e na cobertura universal de saúde ao longo do curso de vida, juntamente com ações sobre determinantes sociais e comerciais estruturais, é essencial para reduzir essa carga.

**Financiamento:**

O Estudo GBD 2023 é financiado pela Fundação Gates.

**Research in context**

**Evidence before this study**

We searched PubMed, Scopus, and the WHO Global Index Medicus (GIM) from inception to Aug 31, 2025, for studies in English, Spanish, or Portuguese using combinations of “oral disorders”, “oral health”, “dental caries”, “periodontal disease”, “edentulism”, “Latin America”, “Caribbean”, “Central America”, “South America”, “disease burden”, “epidemiology”, “prevalence”, and “incidence”. The searches identified 164 records in PubMed, 197 in Scopus, and 63 in GIM. Most non-GBD studies were cross-sectional and condition-specific (chiefly caries in children; fewer on adult periodontitis or tooth loss), with marked heterogeneity in diagnostic criteria (e.g., DMFT, CPI/CPITN variants), sampling frames, and age structures, limiting comparability across countries and time. Evidence was concentrated in Brazil, Mexico, Chile, and Colombia, with additional studies from Argentina, Peru, and Ecuador, whereas evidence from Central America and several Caribbean nations remained sparse. GIM contributed policy and programme reports (e.g., WHO/PAHO country oral health profiles) but few peer-reviewed population studies. Across all sources, we found no long-term projections for oral disorders in Latin America and the Caribbean (LAC). Comprehensive, standardised estimates exist within the Global Burden of Disease (GBD) framework, but prior GBD publications did not provide LAC-specific forecasting for oral disorders.

**Added value of this study**

This study offers the first LAC-wide projections of oral disorders through 2050, based on GBD 2023 estimates (1990–2023) and a transparent forecasting approach. We provide comparable incidence, prevalence, and years lived with disability (YLDs) at country level (33 LAC countries), disaggregated by age and sex, and quantify heterogeneity across subregions. Beyond describing trends, the study contextualizes forecasts within demographic ageing, shifts in age patterns—from childhood caries to adult and older-age disability—and their implications for service demand and oral-health workforce planning. By providing detailed, comparable estimates across the region, this work strengthens the evidence base needed to inform future research agendas and support policy dialogue on oral health in LAC.

**Implications of all the available evidence**

The persistent burden of oral disorders in LAC—particularly the increase in severe periodontitis and edentulism among older adults—calls for a shift from a predominantly paediatric prevention model to a life-course approach that includes adult and geriatric oral health care. Evidence from this study should inform integration of oral health into primary health care, universal health coverage (UHC) schemes, and broader non-communicable disease (NCD) strategies. Moreover, findings of this study should be used for advocacy and mobilization purposes for action on the broader political, commercial and social determinants of oral health in the region.

A critical regional priority is the establishment of robust oral health surveillance systems. With few exceptions, such as Brazil and Colombia, systematic monitoring remains scarce and fragmented. Furthermore, incorporating standardised oral health modules into national health surveys across the region would be a feasible and essential step to generate comparable data, reduce heterogeneity, and track progress towards WHO 2030 oral health targets.

## Introduction

Oral disorders (OD), as defined in the Global Burden of Disease (GBD) framework, include untreated dental caries (UDC), severe periodontitis, edentulism, and other oral conditions.^1^ These disorders impair eating and speaking, and affect social interaction; as they progress, they cause pain and discomfort that affect daily activities, work, rest, and psychosocial wellbeing,^2^ generating substantial disability and economic loss.^3^ In 2021, OD affected an estimated 3.7 billion individuals worldwide,^1^ with a disproportionately higher burden in those populations with limited resources. Despite their preventable nature, OD have historically received limited attention within public health agendas,^4^ and remain among the most prevalent non-communicable diseases (NCDs) globally, generating more than US$700 billion annually in direct and indirect costs.^3^

Against this background of long-standing neglect, in recent years oral health has gained renewed visibility through a coordinated global response. The 2021 World Health Assembly Resolution on Oral Health called for a shift from predominantly curative models to preventive, community-based approaches integrated within primary health care (PHC), and affirmed the inclusion of oral health within NCDs and universal health coverage (UHC) agendas.^5^ In response, WHO developed the Global Oral Health Strategy (2022),^6^ and the Global Oral Health Action Plan 2023–2030,^7^ which set out specific actions for Member States and partners. Momentum was further reinforced in 2024 with the Bangkok Declaration—*No Health Without Oral Health*—which emphasised the need for climate-resilient, equitable, and primary-care-centred oral health systems.^8^

In Latin America and the Caribbean (LAC), this global momentum contrasts with persistent structural challenges that sustain high levels of unmet need and oral health–related disability. Despite overall expansion of health systems, oral health care remains largely fragmented, predominantly curative, and weakly integrated into primary health care, with dental services frequently excluded from publicly funded benefit packages.^9^, ^10^, ^11^ In parallel, health information systems for oral conditions are limited, poorly harmonised, and largely cross-sectional, limiting comparability across countries and age groups, particularly among older adults, who increasingly bear disability related to OD.^1^^,^^11^^,^^12^ Although the region has an overall adequate dental workforce, providers are disproportionately concentrated in urban and private sectors, leaving rural and low-income populations underserved,^11^ with pronounced dental workforce shortages in several Caribbean and Central American nations, including Belize, Guatemala, Honduras, Haiti, and El Salvador.^13^^,^^14^ Financial barriers and high out-of-pocket expenditure further restrict access to preventive and rehabilitative care.^4^^,^^11^ Together, these structural and geographic disparities underscore the need for equity-oriented strategies to strengthen access and service delivery in LAC.

Previous GBD analysis have provided comprehensive, standardised estimates of OD,^1^^,^^15^^,^^16^ but, to our knowledge, none have generated LAC-specific forecasts for these disorders. This gap is particularly relevant in a region undergoing rapid demographic ageing and epidemiological transition, where projections are essential to anticipate service needs and prevent widening inequities.

This study addresses these gaps by presenting a systematic analysis of OD in 33 LAC countries from 1990 to 2023, with projections to 2050. Our objectives are to (1) quantify incidence, prevalence, and years lived with disability (YLDs) across age groups; (2) characterise cross-country heterogeneity and associations with sociodemographic index; and (3) forecast future burden under a reference scenario to inform policy, resource allocation, workforce planning, and advocacy efforts targeting the political, commercial, and social determinants of oral health in the region.

## Methods

### Overview

This study presents estimates from the GBD 2023 for oral disorders in LAC from 1990 to 2023, with projections to 2050.^17^ Within the GBD framework, *oral disorders* are classified at Level 4 into five main causes: UDC of deciduous teeth and permanent teeth, severe periodontitis, edentulism, and other oral disorders.^1^ Definitions were harmonised with the *International Classification of Diseases, 10th Revision (ICD-10)*^18^ and international clinical guidelines to ensure comparability across countries. Analyses follow the Guidelines for Accurate and Transparent Health Estimates Reporting (GATHER) (Appendix 1, Section 2).^19^

### Data sources

GBD 2023 synthesises epidemiological data from multiple sources, including national health surveys, population-based studies, dental examination surveys, administrative health records, and hospital discharge databases across LAC countries (Appendix 1, Supplementary Table S3). Additional data were obtained from PubMed, World Health Organization (WHO) repositories, and national statistical offices. All data sources met GBD inclusion criteria and are publicly available through the Global Health Data Exchange (GHDx).^20^

Across all source types, GBD 2023 stratifies all input data by sex (female and male) and across 25 age groups, spanning from birth to 95 years and older prior modelling. For input data sources that did not report estimates within these standard strata, for example, sources aggregating across both sexes combined or reporting broader age intervals than the GBD five-year age groups, a standardised age-sex splitting method was applied. Observations were redistributed into granular age- and sex-specific data points using reference patterns derived from spatiotemporal Gaussian process regression (ST-GPR) models fitted to data sources that did provide the required disaggregation. Where a source reported data by age but not by sex, within-source sex ratios were applied to produce sex-specific estimates. These splitting methods ensure that all input data, regardless of their original reporting structure, contribute to sex- and age-specific burden estimates in a standardised and internally consistent manner.

Sex-specific estimates were generated for males and females. As no meaningful sex-related differences were identified across the OD in LAC, the primary analyses and figures are presented for both sexes combined.

### Case definition

The GBD project specifies standardised case definitions to ensure consistency and comparability of health outcomes across diverse settings (Appendix 1, Section 3.1). For oral disorders, definitions were based on clinically and pathophysiologically validated criteria from global clinical guidelines, ensuring accurate capture of conditions across data sources.

• Untreated dental caries of deciduous teeth: unmistakable cavity, undermined enamel, softened floor or wall, a tooth with a temporary filling, a filled tooth with recurrent decay, or teeth extracted due to caries (ICD-10 codes K02.0–K02.9, 521.0–521.09).
• Untreated dental caries of permanent teeth: same diagnostic criteria applied to permanent dentition (ICD-10 K02.0–K02.9, 521.0–521.09).
• Severe periodontitis: chronic periodontitis with periodontal pocket depth ≥6 mm and clinical attachment loss ≥5 mm at one or more sites, consistent with CPITN Class IV (ICD-10 K05.0–K05.6, 523.0–523.9).
• Edentulism: complete loss of permanent teeth (ICD-10 K08.1).
• Other oral disorders: a residual, non-specific category comprising a heterogeneous group of dental, tongue, and jaw conditions not captured above (ICD-10 K00–K01.1, K03–K04.99, K07–K08, K08.8–K14.9, M26–M27.9). Consistent with GBD methodological design, oral cancers and congenital malformations are excluded from this category and from the present analysis.

Alternative definitions, such as broader classifications of oral mucosal diseases or non-standard diagnostic thresholds, were excluded to maintain cross-country comparability and focus on high-burden conditions with consistent ascertainment across studies.

Estimates were reported by specific age groups for incidence, prevalence, and YLDs from 1990 to 2023: *UDC of deciduous teeth* in children aged 1–9 years, *UDC of permanent teeth* in individuals aged ≥ 10 years, *severe periodontitis* in individuals aged ≥ 15 years, and *edentulism* in individuals aged ≥ 60 years. Forecasts to 2050 are presented for all ages combined.

### Modelling of incidence, prevalence, and years lived with disability (YLDs)

Nonfatal outcomes, including incidence, prevalence and YLDs for oral disorders, were estimated using the GBD’s standard model-based approaches. Spatiotemporal Gaussian Process Regression (ST-GPR) is used to smooth noisy or sparse time-series data. Measurement error and heterogeneity across epidemiological data sources were further addressed using Meta-Regression—Bayesian, Regularised, Trimmed (MR-BRT), which allows adjustment for systematic reporting biases and inconsistencies, such as differences between self-reported and clinically assessed outcomes.^21^ Where necessary, prevalence input data was also harmonised across alternative case definitions and reporting formats using standard GBD crosswalk procedures to ensure comparability across data sources; full methodological details are provided in the appendix. Subsequently, DisMod-MR 2.1, a Bayesian meta-regression tool, models incidence, prevalence, and disease progression across age, sex, and geography while ensuring internal consistency between epidemiological parameters.^21^ In locations with sparse or no primary data, estimates were informed by patterns observed in geographically and epidemiologically similar locations through the hierarchical structure of DisMod-MR 2.1 and relevant covariates, while uncertainty is propagated through posterior draws.

Within the GBD cause hierarchy, each of the five Level 4 causes of oral disorders was modelled independently in DisMod-MR 2.1. Excess mortality was fixed to zero for all non-fatal OD. Age restrictions were primarily implemented through incidence priors to ensure biological plausibility: incident caries was set to zero in infants younger than 1 year; deciduous caries incidence was constrained to zero from age 11 onwards; and permanent caries incidence was set to zero in children younger than 5 years. Periodontal diseases and edentulism were similarly constrained to zero in early childhood. Prevalence was derived within the compartmental model based on the estimated epidemiological parameters rather than directly truncated by age. Cause-specific estimates were subsequently aggregated to produce Level 3 oral disorder results.

Cause-specific covariates were incorporated where appropriate to improve predictive validity, including lag-distributed income per capita, sugar availability, smoking summary exposure value (SEV) and high fasting plasma glucose SEV, and cardiovascular disease SEV. Covariates were selected based on established epidemiological evidence and model performance criteria. Covariate selection was guided by epidemiological evidence and model performance criteria; detailed specifications, directions of association, and estimated coefficients for each oral disorder are provided in the Appendix 1 (Section 3.2).

Additional methodological details are provided in the Appendix 1 (Section 3).

### Association with socio-demographic development

To examine the relationship between development status and disability burden from OD, we linked age-standardised YLDs rates with the Socio-demographic Index (SDI) for each country in LAC in 2023. SDI is a composite indicator ranging from 0 to 1, where higher values reflect greater development. It is calculated as the geometric mean of three rescaled components: total fertility rate under age 25 years (as a proxy for reproductive health and demographic transition), mean years of educational attainment in the population aged ≥ 15 years (human capital), and lag-distributed income per capita (economic development).^22^ Given the potential for non-linear associations between SDI and YLDs, we evaluated the relationship using *locally weighted scatterplot smoothing* (LOWESS), which is a non-parametric regression technique that fits locally weighted polynomials. Ninety-five percent confidence intervals for the smoothed curves were derived using 1000 bootstrap resamples of the country-level data.

### Forecasts methodology

Projections to 2050 were generated using the GBD reference forecast model. Mixed-effects regression models were first fit to historical age- and sex-specific rates from 1990 to 2021 for each OD, applied to log-transformed rates to stabilise variance and ensure plausible long-term trajectories. These models incorporated country-level random intercepts and covariates reflecting socioeconomic development and key risk exposures, including SDI, sugar consumption, and smoking prevalence. Projected covariate trajectories were then applied to the fitted models to estimate age- and sex-specific rates for 2024–2050 by location. Finally, projected case counts were derived by applying forecasted rates to the corresponding GBD population projections, which incorporate expected changes in fertility, mortality, migration, population growth, and age structure. Thus, projected estimates reflect both changes in epidemiological rates and underlying demographic shifts.^17^ To ensure consistency with the most recent estimation cycle, forecasts were aligned to GBD 2023 estimates at the 2023 baseline (Appendix 1, Section 3.3).

### Calculating uncertainty

All estimates are presented with 95% UIs, defined as the 2.5th–97.5th percentiles of posterior draws. To improve computational efficiency, the number of posterior draws per process was reduced from 500 in GBD 2021 to 250 in GBD 2023. The impact of this reduction was evaluated through simulation analyses that compared point estimates and the coverage of 95% uncertainty intervals across different draw counts. These analyses showed negligible differences in mean estimates and preserved appropriate uncertainty coverage.^21^ Changes over time were interpreted based on the uncertainty intervals of percentage change estimates; a trend was considered unchanged when the 95% UI of the percentage change included zero.

### Ethics approval

This study uses secondary analyses of de-identified, publicly available GBD 2023 data.

### Role of the funding source

The funders of this study had no role in study design, data collection, data analysis, data interpretation, or the writing of the report. The lead and senior authors had full access to the data in the study and final responsibility for the decision to submit for publication.

## Results

**Fig. 1:**
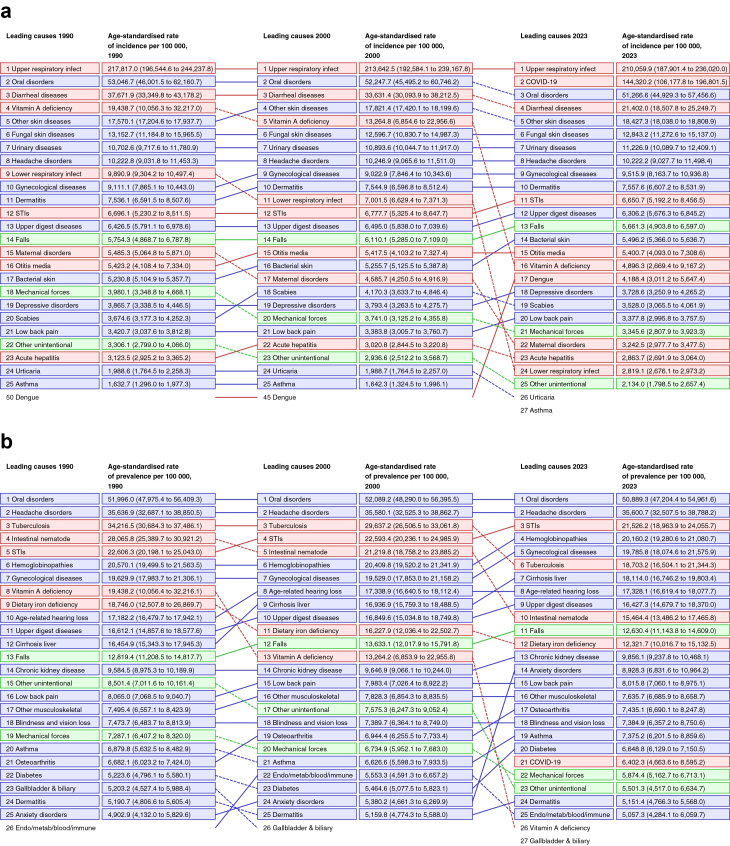

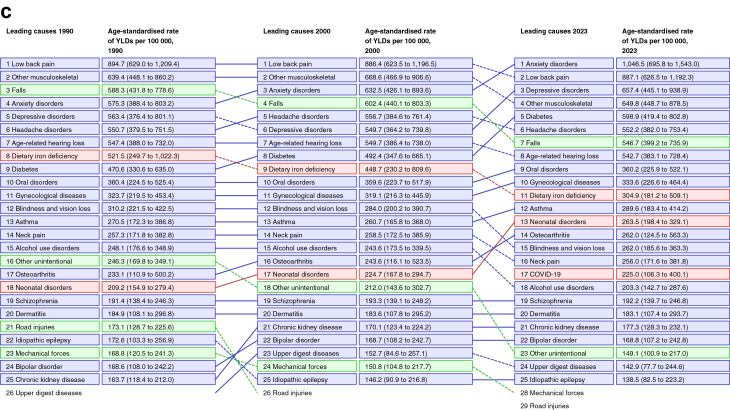
Regional ranking of oral disorders by age-standardised incidence (a), prevalence (b), and YLD (c) rates per 100,000 population in Latin America and the Caribbean, both sexes combined, 1990, 2000, and 2023. YLD = years lived with disability. Colours indicate Global Burden of Disease (GBD) cause groups: blue = non-communicable diseases; green = injuries; red = communicable, maternal, neonatal, and nutritional conditions. Line style: solid lines denote causes that maintained or improved their ranking over time, whereas dotted lines denote causes that declined in ranking.

In 2023, the incidence of OD ranked third among the 25 leading causes of new disease onset in LAC, with an age-standardised incidence rate (ASIR) of 51,266.6 per 100,000 population (95% UI 44929.3–57456.6), comparable to upper respiratory infections and diarrhoeal diseases (Fig. 1A). Oral disorders accounted for 290.8 million new cases (259.3–325.4), representing 8.6% of all incident cases across all causes in the region. Compared with 1990, the ASIR remained stable (−3.4%, −9.0 to 2.2) (Table 1).

**Table 1:** Incidence, prevalence, and YLDs attributable to oral disorders and their subcategories in Latin America and the Caribbean, 2023, with percentage changes in age-standardised rates from 1990 to 2023.

	Incidence			Prevalence			Years Lived with Disability (YLDs)		
	Number of new cases (95% UI)	% of total (95% UI)	Percentage change in age-standardised rate, 1990–2023 (95% UI)	Number of prevalent cases (95% UI)	% of total (95% UI)	Percentage change in age-standardised rate, 1990–2023 (95% UI)	Number of YLDs (95% UI)	% of total (95% UI)	Percentage change in age-standardised rate, 1990–2023 (95% UI)
All GBD causes	3,399,616,147.2 (3,129,835,785.2–3,750,269,013.8)	100		573,761,574.6 (571,643,445.1–575,926,319.0)	100		78,839,661.1 (60,133,735.1–101,286,976.9)	100	
Oral disorders	290,821,803.3 (259,288,629.8–325,444,693.6)	8.6 (7.4–9.6)	−3.4 (−9.0 to 2.2)	308,235,950.8 (285,894,618.3–332,711,390.0)	53.7 (49.9–57.9)	−2.1 (−5.2 to 0.9)	2,410,657.6 (1,513,508.4–3,478,066.2)	3.1 (2.2–4.0)	0.0 (−7.6 to 7.7)
Caries of deciduous teeth	81,997,336.5 (62,617,340.0–102,732,819.6)	2.4 (1.8–3.0)	−7.1 (−20.1 to 10.6)	33,022,321.0 (27,748,437.9–38,282,824.0)	5.8 (4.9–6.7)	−4.6 (−13.6 to 6.9)	12,643.5 (5655.8–25,764.6)	0.0 (0.0–0.0)	−4.9 (−16.5 to 10.1)
Caries of permanent teeth	197,877,770.1 (174,812,188.4–223,610,697.8)	5.8 (5.0–6.7)	−1.4 (−2.7 to 0.3)	197,279,310.4 (172,271,604.6–227,590,197.4)	34.4 (30.0–39.5)	−4.0 (−5.9 to −2.4)	191,402.1 (84,016.2–349,384.6)	0.2 (0.1–0.4)	−4.4 (−6.3 to −2.8)
Severe periodontitis	7,610,233.7 (6,751,078.7–8,531,747.5)	0.2 (0.2–0.3)	0.9 (−11.8 to 19.1)	87,159,569.8 (72,871,018.6–102,457,992.5)	15.2 (12.7–17.8)	3.1 (−16.0 to 31.2)	562,651.8 (220,956.0–1,111,526.1)	0.7 (0.3–1.3)	3.1 (−16.3 to 32.0)
Edentulism	3,336,462.9 (2,961,080.4–3,711,712.8)	0.1 (0.1–0.1)	−2.6 (−11.2 to 10.1)	48,766,441.8 (42,638,025.3–55,369,064.6)	8.5 (7.4–9.7)	−0.5 (−11.3 to 14.0)	1,314,299.0 (873,966.7–1,747,451.6)	1.7 (1.3–2.0)	−0.6 (−11.6 to 13.8)
Other oral disorders				11,536,408.0 (11,057,637.3–11,996,968.2)	2.0 (1.9–2.1)	0.0 (−0.1 to 0.1)	329,661.1 (206,416.2–476,428.4)	0.4 (0.3–0.5)	0.0 (−0.7 to 0.7)

YLD = years lived with disability.

In 1990, 2000 and 2023, the prevalence of OD ranking first among all diseases and injuries in LAC. In 2023, the age-standardised prevalence rate (ASPR) was 50,889.3 per 100,000 population (47,204.4–54961.6), with 308.2 million people affected (285.9–332.7), accounting for 53.7% of all prevalent cases across all causes. Compared with 1990, the ASPR showed no change (−2.1%, −5.2 to 0.9) (Fig. 1B; Table 1). The disability burden was stable across the study period. In 2023, oral disorders ranked ninth for YLDs, with an age-standardised rate of 360.2 per 100,000 population (225.9–522.1), contributing 2.41 million YLDs (1.51–3.48), or 3.1% of total YLDs (Fig. 1C; Table 1).

Subcause analyses revealed heterogeneous patterns across OD (Table 1). UDC of permanent teeth was the leading OD in 2023, with the highest incidence (197.9 million, 174.8–223.6) and prevalence (197.3 million, 172.3–227.6); between 1990 and 2023, its age-standardised incidence rate showed no change (−1.4%, −2.7 to 0.3), while prevalence (−4.0%, −5.9 to −2.4) and YLDs (−4.4%, −6.3 to −2.8) declined modestly. UDC of deciduous teeth had the second-highest incidence (82.0 million, 62.7–102.7) and remained stable across all metrics. Severe periodontitis was the second most prevalent OD (87.2 million, 72.9–102.5), with no changes in prevalence (3.1%, −16.0 to 31.2) or YLDs (3.1%, −16.3 to 32.0). Edentulism accounted for the largest share of YLDs (1.3 million, 0.9–1.7) and showed no change in incidence, prevalence, or YLDs. Other OD also remained stable (prevalence 0.0%, −0.1 to 0.1; YLDs 0.0%, −0.7 to 0.7).

**Fig. 2:**
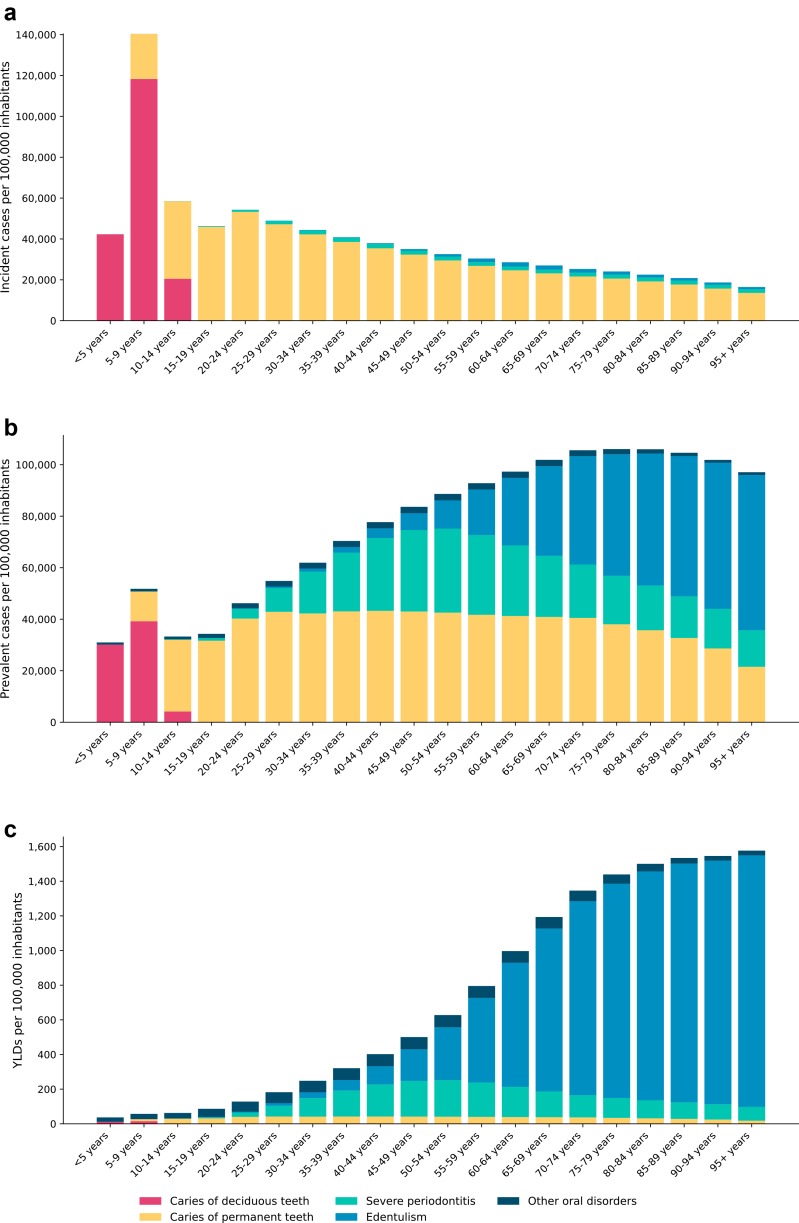
Age-specific incidence (a), prevalence (b), and YLD (c) rates per 100,000 population attributable to oral disorders in Latin America and the Caribbean, both sexes combined, 2023. YLD = years lived with disability.

Age patterns revealed distinct life-course profiles (Fig. 2). UDC of deciduous teeth dominated in childhood, with incidence peaking at 118,287 (95% UI 84900–145,544) per 100,000 at ages 5–9 years, more than double of the peak incidence of UDC of permanent teeth in young adults (53,279 [43,024–62962] per 100,000 at 20–24 years). Prevalence followed a similar transition, deciduous caries predominated in school years, while caries of permanent teeth became the major contributor in adulthood, peaking at 43,255 (28,518–56666) per 100,000 at ages 40–44. Severe periodontitis showed an age-related rise in prevalence beginning in early adulthood, peaking between 40 and 59 years, and declining gradually thereafter. In older ages, the burden increased further as edentulism became more prevalent, reaching 60,249 (52,127–67452) per 100,000. Disability rose sharply with ageing, YLDs rates were minimal for UDC of deciduous teeth (15.0 [6.9–31.4] per 100,000) but rose more than 30-fold with edentulism, peaking at 1451 (1017–1919) per 100,000 in the oldest adults.

**Fig. 3:**
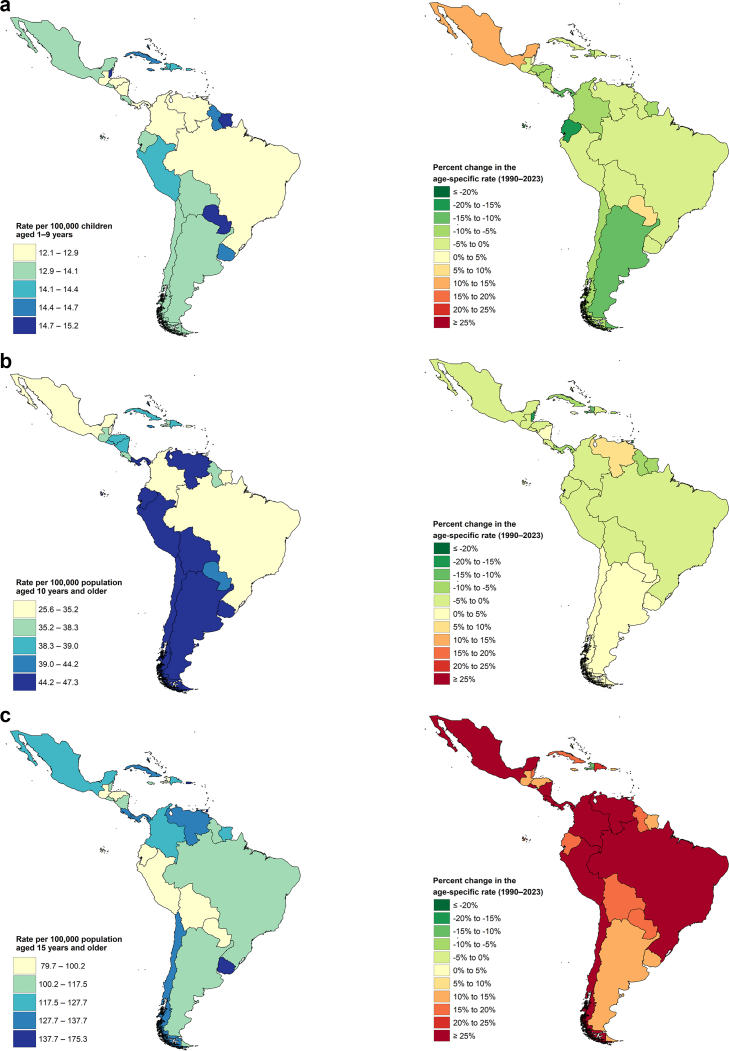

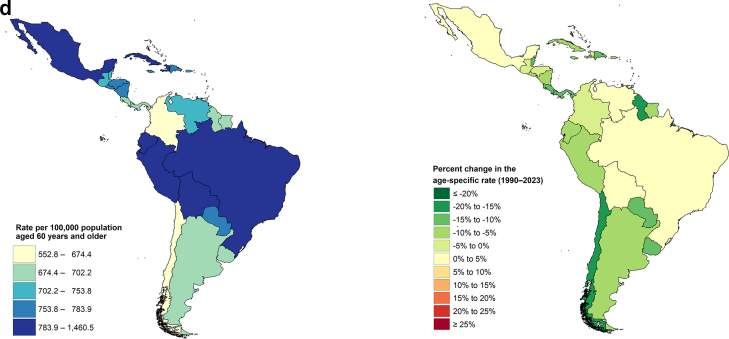
Age-specific YLD rates per 100,000 population of major oral conditions in Latin America and the Caribbean by age group, both sexes combined, 2023. (a) Untreated caries of deciduous teeth among children aged 1–9 years; (b) untreated caries of permanent teeth among individuals aged 10 years and older; (c) severe periodontitis among individuals aged 15 years and older; and (d) edentulism among adults aged 60 years and older. YLD = years lived with disability.

Across countries age patterns also exhibited notable variation in disabilities (Fig. 3). Among children aged 1–9 years, YLDs rates for UDC of deciduous teeth ranged from 12.1 to 15.2 per 100,000 population, with the highest burdens in Belize (15.2 [6.8–29.3] per 100,000) and Paraguay (15.1 [6.8–31.8]), nearly twice those in Brazil and Colombia (<13.0 per 100,000). Between 1990 and 2023, reductions exceeded 15–20% in Chile, Argentina, and Uruguay, whereas Mexico, Paraguay, and several Central American countries showed minimal or no change. Among individuals aged ≥ 10 years, UDC of permanent teeth was highest in Chile (47.3 [23.7–100.9] per 100,000), Bolivia (45.7 [21.9–88.1]), and Ecuador (45.1 [21.4–89.2]), nearly double those in Mexico (25.6 [12.0–49.9] per 100,000). Severe periodontitis (≥15 years) showed the highest YLDs rates in Bermuda (175.3 [97.9–395.6] per 100,000), Puerto Rico (163.9 [85.0–358.9]), and Uruguay (154.7 [82.7–337.4]), nearly twice those in Guatemala and Honduras (<100.0); most countries recorded increases of ≥25% since 1990. Among older adults (≥60 years), edentulism peaked in Bolivia (1460.5 [929.0–1952.8] per 100,000), Peru (1366.9 [809.9–1937.1]), and Brazil (1306.4 [808.6–1751.3]), while Chile and Colombia ranked among the lowest (<680.0), with declines of >15% since 1990.

**Fig. 4:**
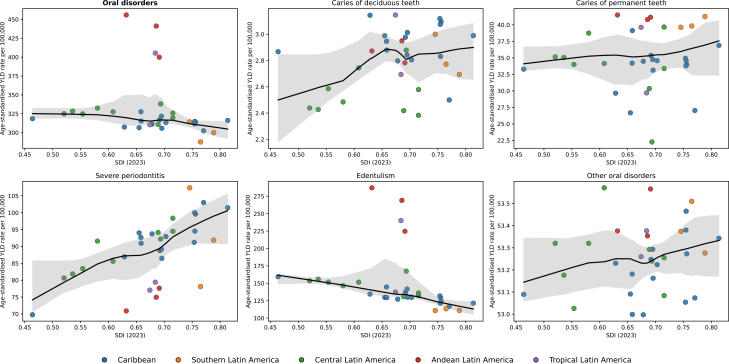
Association between age-standardised YLD rates per 100,000 populations of oral disorders and the Socio-demographic Index (SDI) across 33 countries in Latin America and the Caribbean in 2023. YLD = years lived with disability. Each dot represents one country, coloured by subregion: blue = Caribbean; orange = Southern Latin America; green = Central Latin America; red = Andean Latin America; purple = Tropical Latin America. Solid black lines represent locally weighted regression (LOWESS) curves with bootstrapped 95% confidence intervals (shaded areas).

LOWESS curves revealed heterogeneous associations between SDI and age-standardized YLD rates for OD in 2023 (Fig. 4). Overall, OD showed no consistent gradient across the SDI spectrum. Disability from severe periodontitis increased modestly with higher SDI, whereas edentulism showed a declining pattern, consistent with lower tooth loss in more developed settings. YLD rates for both deciduous and permanent caries remained largely flat across SDI levels, indicating limited variation by development status. Other oral disorders showed no discernible association.

**Fig. 5:**
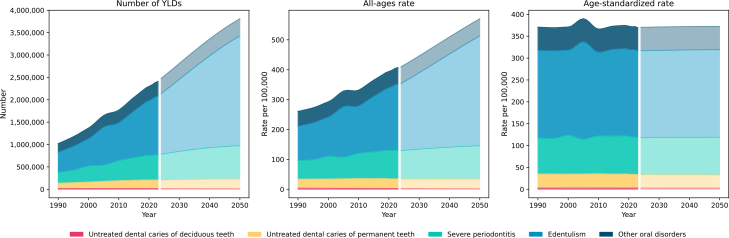
Observed YLD counts and rates per 100,000 population of oral disorders, 1990–2023, and projections to 2050 in Latin America and the Caribbean, both sexes combined. YLDs = years lived with disability. Panels display total YLD numbers, all-age YLD rates per 100,000 population (crude rates), and age-standardised YLD rates per 100,000 population. Solid shading represents observed estimates (1990–2023), and lighter shading represents projected estimates (2024–2050).

Between 1990 and 2023, the number of YLDs nearly doubled, rising from 1.02 million (0.57–1.69) to 2.41 million (1.39–3.71), while age-standardised rates remained broadly stable. Projections to 2050 anticipate a rise to about 3.81 million YLDs (2.28–5.80) and an age-standardised rate of 374 per 100,000 (248–532). Subcause projections show slight declines in UDC of deciduous teeth, gradual increases in permanent teeth and severe periodontitis, and edentulism persisting as the largest contributor to disability (Fig. 5).

## Discussion

This study provides the first comprehensive, standardised assessment of oral disorders in LAC over three decades, with projections to 2050 based on robust data from GBD 2023 Study. Three key findings stand out. First, despite modest declines in incidence, age-standardised prevalence and disability have remained broadly stable since 1990, underscoring the limited impact of current prevention strategies and the persistent neglect of oral health within the NCDs agenda. Second, the burden is heterogeneous, with declines in UDC of deciduous teeth in Southern countries, stable burdens of UDC of permanent teeth and edentulism in Central America, Brazil, and Mexico, and rising burdens of severe periodontitis in most countries. These findings reflect structural inequalities between countries and align with evidence that oral health is determined by the same social and commercial determinants shaping other NCDs.^3^^,^^23^^,^^24^ Third, demographic ageing will substantially increase the number of people living with disability, with edentulism and severe periodontitis acting as dominant drivers and reflecting weakly structured health services and a long-standing curative orientation.^25^ Together, these findings reinforce the need to embed oral health in primary health care (PHC) and universal health coverage (UHC) through a life-course approach with measurable 2030 targets.^7^^,^^11^

Country trajectories appear closely linked to access to preventive measures. Were fluoride toothpaste is affordable and preventive screening is routine, national burdens are lower,^13^^,^^26^^,^^27^ reflecting gains achieved through sanitation, fluoridation, and school-based programmes since the 1980s.^28^ However, fluoride-centred strategies, effective for childhood caries, now reach most of the population via toothpaste, water, or salt fluoridation (e.g., Brazil, Chile, Mexico, Colombia, Peru), yet adult burden remains high. This points to the limits of focusing predominantly on fluoride while overlooking broader determinants such as diet, tobacco use, and access to rehabilitative care. These gaps are most evident among socially excluded, rural, and Indigenous populations who remain underserved by both preventive and restorative services.^29^

This pattern aligns with the common risk factor framework, recognising that oral diseases share behavioural, environmental, and commercial determinants with other NCDs.^29^^,^^30^ Upstream drivers, including poverty, unequal educational opportunities, precarious employment, food environments shaped by powerful commercial interests, and weak PHC capacity, interact over the life course to shape oral health trajectories. Addressing them requires coordinated intersectoral action that links oral health with social protection, education, agricultural and fiscal policies, while strengthening oral health surveillance and oral epidemiology to close persistent data gaps and support accountability.^31^, ^32^, ^33^

Regional progress requires moving beyond a predominantly clinical paradigm toward integrated approaches that combine prevention, public health action, and improved access to essential oral health care in LAC. Without tackling these upstream determinants, investments in service expansion and workforce training will have limited impact on equity and disability. Advancing towards WHO 2030 oral health targets will therefore require aligning oral health policy with broader agendas on NCDs, healthy ageing, and the reduction of social and commercial inequities.

Policy and system responses should focus on three complementary domains: (1) strengthening prevention through population-wide measures that address shared NCD risk factors; (2) integrating oral health into PHC and UHC benefit packages to ensure continuity of care across the life course; and (3) advancing fiscal and governance reforms, including taxes on sugar-sweetened beverages and tobacco with earmarked revenues, and payment mechanisms that incentivise prevention. Within this agenda, research and academic institutions are essential to generate robust data, monitor progress toward 2030 targets, and evaluate the impact of interventions to guide evidence-based policy and resource allocation.

This study has limitations inherent to the GBD framework. First, country-level data availability is uneven, fourteen of the 33 LAC countries (predominantly Caribbean locations) had no primary data sources for OD, and several others had only partial or outdated inputs, leading to substantial heterogeneity in the empirical support behind estimates. In settings with sparse or absent data, results rely more heavily on statistical modelling and covariate patterns, which is reflected in wider uncertainty intervals. Second, health information systems for oral conditions are fragmented and largely cross-sectional, limiting comparability across countries, age groups, and survey instruments, and constraining the assessment of cumulative disability across the life course. Third, for input sources that did not report data disaggregated by sex or within standard GBD five-year age groups, sex- and age-specific estimates were derived through statistical splitting methods using reference patterns from other data sources rather than direct measurement, increasing uncertainty, particularly in settings and age groups with limited empirical data. Fourth, GBD estimates edentulism as complete tooth loss and does not capture partial edentulism. This methodological choice may lead to underestimation of the true burden of tooth loss, particularly among adults aged 60 years and older, among whom partial tooth loss is highly prevalent. Missing teeth—even in the absence of complete edentulism—can result in substantial functional impairment, including reduced masticatory efficiency, nutritional limitations, and loss of original functional capacity, which are not fully reflected in current GBD estimates. Fifth, projections assume continuity of current trends in risk factors and health system performance; abrupt policy, technological, or socioeconomic shifts could substantially alter trajectories. Finally, disability weights may not fully capture the cultural and social valuation of conditions such as edentulism in LAC, where tooth loss can have profound implications for nutrition, communication, and social participation.

Future research in LAC should prioritise improving the coverage, comparability, and continuity of oral health data through standardised national surveys that include adults, older adults, and populations frequently excluded from routine monitoring. Oral health metrics should be systematically integrated into routine health information systems and NCDs surveillance to enable life-course monitoring and strengthen equity accountability. Future burden assessments should also incorporate additional conditions affecting the oral and craniofacial region—such as lip and oral cavity cancer and orofacial clefts—to better characterise the broader spectrum of orofacial disease burden. Analyses across the GBD subregions of LAC could further help capture intra-regional variability in burden of OD and inform more context-sensitive policy responses.

Key evidence gaps remain regarding scalable models for delivering preventive and rehabilitative oral health care within PHC and UHC frameworks. Robust evaluation is needed on the effectiveness and equity impacts of fiscal policies, universal dental benefit packages, and workforce task-sharing strategies. Upcoming GBD cycles should include key oral health risk factors—such as sugar intake, tobacco use, and inadequate fluoride exposure—to better inform prevention and policy.

Building regional capacity in implementation science, health economics, and equity-focused analysis, alongside durable collaboration among ministries of health, academia, and PAHO/WHO, is essential to translate evidence into sustained, context-appropriate action.

### Conclusion

OD remain a leading cause of disability in LAC and have ranked first in prevalence for three decades, underscoring that current approaches are not working. Although age-standardised rates have changed little, population ageing will increase the absolute burden of disability, with edentulism persisting as a major contributor. Without full integration of oral health into PHC and UHC, the region will carry a growing and largely avoidable disability burden into 2050.

Reaching WHO 2030 targets requires a dual shift: strengthening life-course, person-centered prevention and early detection alongside timely rehabilitative care, supported by workforce, financing, and governance; and acting on the upstream social and commercial determinants that shape sugar intake, tobacco exposure, and access to fluoride. Progress should be assessed by continuity and equity of care from childhood to older age, rather than by episodic coverage alone.

## Contributors

Detailed information on individual author contributions is provided in Appendix 2 (p 3). Contributions are reported across the following categories: management of the overall research enterprise; drafting of the initial manuscript; application of analytical methods to generate estimates; data acquisition, cataloguing, extraction, and cleaning; development of figures and tables; provision or critical assessment of data sources; development of methods or computational infrastructure; critical review of analytical methods or results; drafting or critical revision of the manuscript for important intellectual content; and management of the estimation or publication process. The lead and senior authors had full access to the data in the study and final responsibility for the decision to submit the manuscript for publication.

## Data sharing statement

This study adheres to the Guidelines for Accurate and Transparent Health Estimates Reporting (GATHER). Citations and metadata for the data sources used in the Global Burden of Disease (GBD) 2023 analyses presented here are publicly available through the GBD 2023 Sources Tool (https://ghdx.healthdata.org/gbd-2023/sources).

## Declaration of generative AI and AI-assisted technologies in the manuscript preparation process

Artificial intelligence–based tools, including Google Gemini and ChatGPT, were used to support translation from Spanish into English and to improve edition in specific sections. All text assisted by these tools was critically assessed and edited by the authors, who retain full responsibility for the accuracy, originality, and integrity of the final version.

## Editor note

The Lancet Group takes a neutral position with respect to territorial claims in published maps and institutional affiliations.

## Declaration of interests

CH reports employment and a contractual relationship with the Pan American Health Organization (PAHO). RN reports a leadership role as a board member of the American College of Cardiology and receipt of materials from GC America. MC reports leadership roles as President of the Civil Society Advisory Council of the Chilean Ministry of Health for Tobacco Control and as National Treasurer of the Chilean College of Dentists. All other authors declare no competing interests.
