# Supplementary material for: The burden of oral disorders in Latin America and Caribbean countries from 1990 to 2023 and projections until 2050: a systematic analysis for the Global Burden of Disease Study 2023
Source: Lancet Reg Health Am. 2026 Jun 8;60:101517. doi: 10.1016/j.lana.2026.101517 (PMC13264252; doi:10.1016/j.lana.2026.101517)
Supplement: Appendix 1 and 2 [file mmc1.docx]

**Supplementary appendix 2**

**The burden of oral disorders in Latin America and Caribbean countries from 1990 to 2023 and projections until 2050: a systematic analysis for the Global Burden of Disease study 2023**

This appendix provides further authorship detail for “*The burden of oral disorders in Latin America and Caribbean countries from 1990 to 2023 and projections until 2050: a systematic analysis for the Global Burden of Disease study 2023”*.

**Table of contents**

[**GBD 2023 Latin America and Caribbean Oral Disorders Collaborators** 3](#_Toc227879856)

[**Affiliations** 3](#_Toc227879857)

[**Authors’ Contributions** 4](#_Toc227879858)

[Management of the overall research enterprise 4](#_Toc227879859)

[Drafting of the initial manuscript 4](#_Toc227879860)

[Application of analytical methods to generate estimates 4](#_Toc227879861)

[Data acquisition, cataloguing, extraction, or cleaning; development of figures and tables 4](#_Toc227879862)

[Provision of data or critical assessment of data sources 5](#_Toc227879863)

[Development of methods or computational infrastructure 5](#_Toc227879864)

[Critical review of analytical methods or results 5](#_Toc227879865)

[Drafting or critical revision of the manuscript for important intellectual content 5](#_Toc227879866)

[Management of the estimation or publication process 5](#_Toc227879867)

# **GBD 2023 Latin America and Caribbean Oral Disorders Collaborators**

Maria Jesus Rios-Blancas, Christian Razo, Linda Morales-Juárez, Ricardo X. Martinez, Lucero Lopez-Lopez, Fernando Neves Hugo, S. Aida Borges-Yáñez, Carol Guarnizo-Herreño, Carolina Hommes, Caroline Stein, Betine P. Moehlecke Iser, Richard Niederman, Escoffié-Ramírez Mauricio, Vanessa Aldaz-Rodriguez, Roberto A. Leon-Manco, Deborah Carvalho Malta, Marco Cornejo-Ovalle, Carlos Garcia-Zavaleta, Paul Nam, Rafael Lozano*.

*Senior author.

# **Affiliations**

Department of Public Health, School of Medicine, National Autonomous University of Mexico, Mexico City, Mexico, Carlos Slim Foundation (M Rios-Blancas PhD); Institute for Health Metrics and Evaluation, University of Washington, Seattle, Washington, United States of America, Department of Health Metrics Sciences, School of Medicine, University of Washington, Seattle, Washington, United States of America (C Razo PhD); Department of Public Health, School of Medicine, National Autonomous University of Mexico, Mexico City, Mexico (L Morales-Juarez PhD); Sensory and Oral conditions, Noncommunicable Diseases and Mental Health, World Health Organization (R Martinez PhD); Octoma, Tequisquiapan, Mexico (L Lopez-Lopez PhD); Department of Epidemiology and Health Promotion, New York University College of Dentistry, United States of America (F Neves PhD); Facultad de Odontología, Universidad Nacional Autónoma de México, Ciudad de México, México (A Borges-Yañez PhD); Facultad de Odontología, Universidad Nacional de Colombia, Colombia (C Guarnizo-Herreño PhD); International Consultant, Pan American Health Organization / World Health Organization (C Hommes MPH); Institute for Health Metrics and Evaluation, University of Washington, Seattle, Washington, United States of America (C Stein PhD); Postgraduate Program in Health Sciences, University of Southern Santa Catarina, Brazil (B Moehlecke PhD); Department of Epidemiology & Health Promotion, New York University College of Dentistry, United States of America (R Niederman DMD); School of Dentistry, Autonomous University of Yucatan, Mexico (M Escoffie-Ramirez DPH); Open and Distance Education System, School of Medicine, National Autonomous University of Mexico, Mexico City, Mexico (V Aldaz-Rodriguez MHSA); Faculty of Dentistry, Universidad Peruana Cayetano Heredia, Peru (R Leon-Manco MPH); Escola de Enfermagem, Universidade Federal de Minas Gerais, Brazil (D Carvalho PhD); Department of Public Health, Faculty of Dentistry, University of Chile, Santiago, Chile (M Cornejo PhD); Faculty of Dentistry, Universidad Peruana Cayetano Heredia, Peru (C Garcia-Zavaleta DDS); Institute for Health Metrics and Evaluation, University of Washington, Seattle, Washington, United States of America (P Nam Bs); School of Medicine, Universidad Nacional Autónoma de México, Mexico, Institute for Health Metrics and Evaluation, University of Washington, Seattle, Washington, United States of America (Prof R Lozano MD).

# **Authors’ Contributions**

## Management of the overall research enterprise

Rafael Lozano, Maria Jesus Rios-Blancas, Christian Razo, Linda Morales-Juárez.

## Drafting of the initial manuscript

Maria Jesus Rios-Blancas, Rafael Lozano.

## Application of analytical methods to generate estimates

Paul Nam, Christian Razo.

## Data acquisition, cataloguing, extraction, or cleaning; development of figures and tables

Paul Nam, Christian Razo, Maria Jesus Rios-Blancas.

## Provision of data or critical assessment of data sources

Paul Nam, Christian Razo, Maria Jesus Rios-Blancas.

## Development of methods or computational infrastructure

GBD 2023 Oral Disorders Collaborators, Paul Nam, Christian Razo.

## Critical review of analytical methods or results

Rafael Lozano, Maria Jesus Rios-Blancas, Christian Razo, Linda Morales-Juárez, Ricardo X. Martinez, Lucero Lopez-Lopez, Roberto A. Leon-Manco, Carol Guarnizo-Herreño, Fernando Neves Hugo, Carolina Hommes, Betine P.Moehlecke Iser, Richard Niederman, Deborah Carvalho Malta, S. Aida Borges-Yáñez, Carlos Garcia-Zavaleta, Caroline Stein, Marco Cornejo-Ovalle, Escoffié-Ramírez Mauricio, Vanessa Aldaz-Rodriguez.

## Drafting or critical revision of the manuscript for important intellectual content

Rafael Lozano, Maria Jesus Rios-Blancas, Christian Razo, Linda Morales-Juárez, Ricardo X. Martinez, Lucero Lopez-Lopez, Roberto A. Leon-Manco, Carol Guarnizo-Herreño, Fernando Neves Hugo, Carolina Hommes, Betine P.Moehlecke Iser, Richard Niederman, Deborah Carvalho Malta, S. Aida Borges-Yáñez, Carlos Garcia-Zavaleta, Caroline Stein, Marco Cornejo-Ovalle, Escoffié-Ramírez Mauricio, Vanessa Aldaz-Rodriguez, Paul Nam.

## Management of the estimation or publication process

Rafael Lozano, Maria Jesus Rios-Blancas, Christian Razo.
